# College Students with Inflammatory Bowel Disease: A Qualitative Study of Challenges Associated with College Transition and Self-Care

**DOI:** 10.1089/heq.2019.0053

**Published:** 2020-05-12

**Authors:** Naueen A. Chaudhry, Angela Pham, Andrew Flint, Isaac Molina, Zareen Zaidi, Ellen M. Zimmermann, Linda S. Behar-Horenstein

**Affiliations:** ^1^ Division of Gastroenterology, Department of Medicine, College of Medicine, University of Florida, Gainesville, Florida, USA.; ^2^ Division of General Medicine, Department of Medicine, College of Medicine, University of Florida, Gainesville, Florida, USA.; ^3^ Educational Development and Evaluation, CTSI, University of Florida, Gainesville, Florida, USA.

**Keywords:** college students, college transition, focus group, inflammatory bowel disease

## Abstract

**Introduction:** The social impact of inflammatory bowel disease (IBD) on student transition to college is significant, yet poorly understood.

**Methods:** Two 90-min focus groups (FGs) were conducted with eight student-patients with IBD. Reflective journals were used to corroborate, elaborate, or challenge emergent FG findings.

**Results:** Six themes emerged: (1) transitioning to college, (2) interacting with physicians, (3) affecting social life, (4) managing the disease by yourself and through support, (5) coping strategies, and (6) facing disease challenges. These themes remained relevant in the reflective writings. Analysis of serial journal entries showed that students' social life and engagement in coursework was affected 66% and 54% of the time, respectively.

**Conclusion:** Our findings offer guidance for improving students' college success, quality of care, and enhancing physician–patient interactions. Students with IBD have a disability that may not be obvious or visible. They require specific support to help them transition and succeed in college.

## Introduction

Inflammatory bowel disease (IBD) is an umbrella term comprising Crohn's disease (CD) and ulcerative colitis (UC). The onset of IBD is typically in the second or third decade of life; the diseases are lifelong and incurable. The prevalence of IBD in adults in the United States is estimated to be 2–4 per 1000 population.^1^ Recent disease trends show an increase in both UC and CD particularly among individuals aged under 20 years.^2^ While the prevalence of IBD on college campuses is not known, data suggest that ∼67,000 student–patients are coping with IBD at college campuses nationally.^3^ A chronic disease of this nature that affects the gastrointestinal tract causing troubling daily symptoms and requiring medications, monitoring, and medical testing is a challenge for all patients. Perhaps no more challenging period exists than when a patient enters college.

**FIG. 1.**
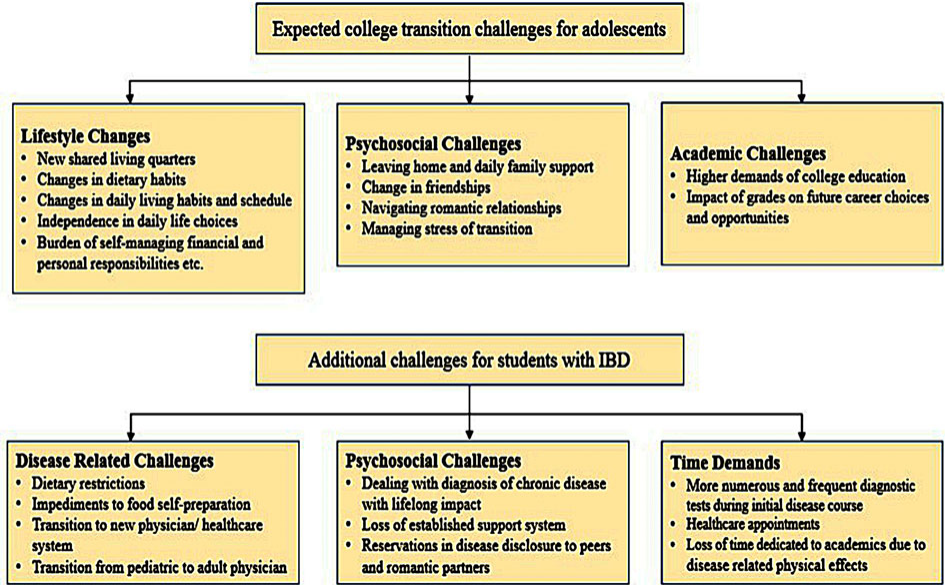
Challenges of college transition faced by students with and without IBD. IBD, inflammatory bowel disease.

For all students, the transition to college life requires a variety of adjustments^4^ (Fig. 1). Adjusting to college is dependent on conceptual categories describing student transition, acclimation, and induction.^5^ For the adolescent who lacks a support network, this process is often challenging.^6^ Entrance into the college setting often presents academic and interpersonal challenges for students as they learn to negotiate shared living quarters, increased educational demands, new social and dating situations, leaving home, and sudden independence. Previous studies have shown that this period is particularly demanding for students with disabilities and for those suffering from chronic illness although little is known about measures of effective assistance.^6,7^ Prior work has shown that IBD exerts a crucial impact on the college experience.^8^ Conversely, attending college can affect the disease. Attending college can cause an interruption in health care either by relocation or by transitioning from a pediatric to a new adult health care provider. Changes in living situation, lifestyle, diet, and stress can all affect disease status. For students with IBD, there may be a disruption in previously established dietary habits, medication, and hygiene regimens. Efforts to settle in, make new friends, and do well in college are likely to be more urgent to students than being diligent about their health care needs.^7^ A preliminary study of students with IBD at the University of Michigan by Adler et al.,^9^ and a subsequent national study by Almadani et al.,^8^ found that college adjustment is strongly associated with disease activity. To ensure that college-age students experience a smooth transition from pediatric to adult IBD care, Gray et al.^10^ recommended collaboration among physicians, parents, and students. The aim of this research is to comprehensively understand the social challenges, coping mechanisms, and academic challenges that students with IBD face.

## Methodology

After receiving institutional review board (IRB201600191) approval, purposive sampling was undertaken to recruit participants. Flyers were posted on campus and in clinics. Of the 50 students with IBD interested in research, 8 were able to attend the focus groups (FGs) at a mutually convenient time. Study participants signed an approved informed consent form before commencement of the first FG session. After receiving participant permission, the proceedings of both FG sessions were audio recorded and later transcribed. Pseudonyms were assigned to participants to ensure their anonymity. Participants were given $50 gift cards per session for their time and were served dinner. Table 1 provides characteristics of the study participants.

**Table 1.** Demographic, Disease Related, and Academic Variables for Focus Group Participants

Variables	Total (*n*=8), *n* (%)
Age, mean (±SD)	20.38 (±2.5)
Gender	
Male	3 (37.5)
Female	5 (62.5)
Race	
Caucasian	6 (75)
Hispanic	2 (25)
Age at IBD diagnosis, mean (±SD)	16.6 (±4.34)
Type of disease	
Crohn's disease	5 (62.5)
Ulcerative colitis	3 (37.5)
Education level	
Undergraduate	6 (75)
Graduate	2 (25)
No. of class credits in current semester	
<6	0
6–12	2 (25); graduate^*^
>12	6 (75), undergraduate^*^
Moved out of town for college	
Yes	8 (100)
No	0
Moved out of state for college	
Yes	0
No	8 (100)
Living accommodations	
With family	0
On campus	4 (50)
Off campus	4 (50)
Education interrupted due to IBD	
Yes	1 (12.5)
No	7 (87.5)
Dropped a class due to IBD	
Definitely	2 (25)
Somewhat	1 (12.5)
Not really	1 (12.5)
Not at all	4 (50)
Participation in team athletics	
Yes	3 (37.5)
No	5 (62.5)
Gastroenterologist location	
Local	3 (37.5)
Hometown	2 (25)
Both local and in hometown	3 (37.5)
Type of gastroenterologist	
Pediatric	2 (25)
Adult	6 (75)
College education funded by	
Scholarship	1 (12.5)
Parental/family support	5 (62.5)
Student loans	1 (12.5)
Employment	1 (12.5)

^*^ Represents the correlation of the education level of the participants with the credit hours enrolled.

IBD, inflammatory bowel disease; SD, standard deviation.

### Data collection

We used two FG discussions 90 min each to explore our research questions. An interview guide of questions (Table 2) was developed, and questions were sent to the participants before the scheduled FG to encourage reflection about their experiences with IBD. Overall the intent of the first FG was to provide an in-depth, contextually rich understanding of phenomena that accrues from participants' words to explain “why” something occurred, not “what” resulted. The second FG allowed member checking of the emergent themes from the first FG and provided support for the themes.

**Table 2.** Focus Group and Weekly Reflective Journal Questions for College Students with Inflammatory Bowel Disease

Focus group questions
1. How has living with IBD influenced planning your transition to college?
2. Describe the guidance that your physician and others provided before your transition. Please provide examples
3. Discuss what, if anything, your physician offered that was the most important advice for your transition
4. Discuss what, if anything, important advice others in your life offered that was the most important for your transition
5. How has IBD impacted your social life during the transition? Provide examples
6. How has having IBD influenced your interest and or involvement in college extracurricular activities?
7. How do you believe IBD will impact (or has impacted) your college social life?
8. During this transition period, what coping mechanisms have you used during your most difficult days with IBD?
Prompts for weekly reflective journals
1. Describe how IBD affected your social life (this week)
2. Describe how you coped with the challenges of IBD (this week)
3. Describe how IBD affected your ability to engage in coursework (this week)

### Reflection papers

A secondary dataset, reflective journals, was used to corroborate, elaborate, or challenge findings that emerged from the FGs. Participants were asked to respond to and write up to 500 words to three researcher-constructed prompts per week (Table 2) and describe how IBD affected their social life and coursework and what challenges it presented.

### Analysis of FGs

All interviews were audiotaped and later transcribed for analysis. We analyzed the compiled data as described ahead by applying grounded-theory approach as described by Charmaz^11^ to identify any unique themes that arose from the collected data. Two authors (N.A.C. and L.S.B.H.) independently open coded the data. Next, they met to categorize their open codes and identified emergent themes. Creswell's^12^ strategies: triangulation, thick, rich description, clarifying researcher bias peer reviewer, and an audit trail were used to enhance the rigor of the data collection and analytical process (Table 2). Using line-by-line coding, initial coding, and focused coding, each FG transcription was analyzed as a separate entity. The initial codes were tested against extensive data using the constant comparative method. Some codes were moved accordingly to better fitting codes or to other categories. Some themes coalesced, while others expanded in the process.

### Analysis of reflection papers

All of the participants completed reflection papers for 6 weeks as requested by the researchers, resulting in a total of 48 journal entries. Two of the authors (N.A.C. and L.S.B.H.) analyzed reflective journal writings to determine the frequency at which participants' social lives and coursework were affected by IBD.

## Results

### Focus groups

Six main themes were identified as representational of college-aged students with IBD: transitioning to college, interacting with physicians, affecting social life, managing the disease by yourself and through support, coping strategies, and facing disease challenges (Table 3). The students participating in the study had been diagnosed greater than at least 1 year, were not undergoing a flare of IBD, and were already on established treatment regimens. At the time of the study, two students were on dual therapy with an immunomodulator and a biologic, four were on monotherapy with a biologic, and the remaining two were off therapy at the time. All of them were off steroids. Two of the students reported having previous bowel surgery for IBD (one off treatment after total colectomy for UC). The FG revealed the considerable struggles and challenges that participants faced while trying to experience the “college life.”

**Table 3.** Emergent Focus Group Themes, Conceptual Definition, and Representative Excerpts Among College Students with Inflammatory Bowel Disease

Themes	Conceptual definitions	Representative narrative
1. Transitioning to college	Preparing to manage IBD independently in a new environment	“A big thing that I thought was like the proximity of how close my choice of university would be to my pediatric gastroenterologist”
2. Interacting with physicians	Communications that aided or impeded disease management	“What my physician has kind of guided me to do, is more quantity versus quality. You don't eat a huge meal, because that's often harder to digest than if you eat five smaller meals a day.”
		“Then I came here a week later, found a physician here, who just really scared me.”
3. Affecting social life	Limitations in socializing	“It just changes what I do, so like one of my best friends and I use to always work out, before I got diagnosed.”
4. Managing the disease by yourself and through support	Personal choices, family, and physicians that aided coping with IBD	“I just learned that I had to budget my time much better than I did.”
5. Coping strategies	Self-care techniques that lessened impact of the disease	“So I let myself sleep extra if I need to sleep and I don't feel guilty about it… just do things that I typically wouldn't have done before.”
6. Facing disease challenges	Patients and their social circle's coping with the expected and unexpected facets of IBD.	“I expected there would be challenges just to have the freedom to eat whatever you want, as a college student.”
a. Taking time for family/friends to adjust	a. Friends and family need information to better understand patients' experiences	“Between the food and going to the doctor, everything from the moment I got diagnosed to now was unexpected.”
b. Disease challenges unexpected	b. Unanticipated ways that the disease impacted patient lifestyle	

#### Transitioning to college

Students who were diagnosed with IBD closer to, or after, transition to college reported greater difficulty making adjustments to college life compared to those diagnosed in early adolescence. Participants described being overwhelmed by both the demands of college adjustment, as well as those of their new diagnosis of chronic disease. The emotional struggles of coming to terms with their diagnosis were an added burden.

Amy described the challenges of balancing school and IBD:

“Between school and trying to keep track of everything, everything was so new to me that it was pretty hard to keep up with everything…. I actually had to leave school at the end of the semester and kind of go home and regroup.”

Students who were diagnosed in early adolescence or before college transition had the benefit of having awareness of their IBD needs and guidance from their physicians and family. Students discussed that college faculty were not empathic of their disease-related needs. Several students mentioned that their middle or secondary school teachers were more accommodating than college professors. Joan stated:

“I noticed the professors were not rude, but they weren't understanding or informed about how to deal with colitis… when I had to miss class for treatments, they give me a hard time about it. So, I didn't expect that kind of reaction when in middle school and high school, for the most part, it was handled a bit better.”

When considering which college they hoped to attend, students factored in the nature of college accommodations and personal needs into transition. They talked about the importance of having private bathrooms to avoid social embarrassment. Since most students had dietary restrictions, they also preferred having cooking amenities as dining hall food could set off a “flare.”

#### Interacting with physicians

All but one student held greater value in their physician's expertise over communication skills although they all appreciated effective communication regarding disease and disease management. Two participants changed physicians twice because they were not satisfied with their communication. Amy reported:

“I am on my third doctor and I got diagnosed a year and a half ago. I had some just not very great experiences with the physicians. The first one diagnosed me, gave me a pamphlet, and sent me on my way.”

Students also reported having inconsistent advice about diet from physicians, which was sometimes a source of frustration. Larry explained the detailed advice he received.

“My physician told me to at least try a low fiber diet and then whenever you go out with friends, just try sparingly what you're eating and don't go all out and see how you take it the next couple of days and then make your judgements there regarding what you can go for.”

Three of the students explained that their physicians offered guidance about the importance of managing college stress. Craig noted:

“[My physician] also emphasized cutting down stress in any way possible and maintain exercise routines even though I will be busy.”

Most students were advised by their physicians that they should test their limits for alcohol since going to bars or drinking during social events was likely in their new college environment.

#### Affecting social life

The majority of the social limitations, which students experienced in the college environment, centered upon diet and alcohol. Since most social interactions with their peers involved going out for food or drinks or joining a sorority or club, they were often faced with the conundrum of either declining these opportunities or facing awkward explanations regarding their dietary choices. All described personal conflicts regarding the decision to divulge their illness to others. They expressed guilt when others made special accommodations for their condition. Others improvised so they could still enjoy the company of others without suffering personally, for example, Frank opted to be the designated driver. They felt that the number and quality of their social interactions and hence their circle of friends were impacted by IBD and its daily nuances, causing occasional anger and irritation.

Amy and Mary talked about the difficulties of dating while having dietary and alcohol limitations due to IBD. Amy described an initial negative impact on her relationship with her fiancé when they couldn't enjoy restaurant dinners and social occasions as often, but they grew closer after they managed to work through the hardships together.

#### Managing the disease by self and through support

The students' habits spoke of self-discipline in several aspects of their lives, including medications, diet, alcohol, caffeine, and physical activity. In general, they tried to maintain a low residue and low fiber diet. A liquid diet and fasting were also necessary sometimes when they experienced disease “flares.” Some of the students resorted to doing their own dietary experimentation such as attempting certain foods and adding anti-inflammatory supplements such as curcumin.

More than one student commented that they had learned to “listen” to their bodies and make accommodations. Mary, a former college athlete, adjusted her regular exercise routine due to periods of low energy. Joan chose not to apply to certain student leadership positions given her struggles with IBD and classes. The students allowed for extra rest and relaxation techniques when they felt the physical need for it.

More than one student talked about how their mothers had helped them navigate the journey to understand their disease and its impact. Frank shared that:

“My mom always taught me after I was diagnosed to budget time for myself and just like have some time in each day where I can just, you know, de-stress and relax and just you know think about myself and my life and all that.”

Students became advocates for themselves during the course of managing their disease. They overcame their shyness and become forthright about voicing their questions and concerns to their physicians. Slowly, they figured out the “right” questions to ask physicians with regards to their IBD. Larry met with children and teens newly diagnosed with IBD upon the request of their parents, to offer advice and insight on dealing with the disease. Frank became a more prominent advocate by speaking at events for CD and plans to continue in this role.

#### Coping strategies

All students unanimously endorsed the importance of stress relief. They referred to individual methods of stress relief such as exercise, yoga, extra sleep, keeping to-do lists, budgeting alone time, listening to rap music, aromatherapy, and doing art. They also validated the association between rising stress and poorly managed symptomatic IBD. Larry explained that:

“I just lay down and put headphones on. I have this soundtrack that's awesome to just mellow you out…. Sometimes I try and fall asleep on it.”

In addition to dietary and alcohol restrictions they were advised to avoid an excessive intake of coffee. Three participants discussed the gastrointestinal side effects of coffee and their adaptations to satisfy cravings (e.g., espresso shots, black and matcha tea, and caffeine pills).

Frank avoided watching TV due to enticing food commercials, whereas Jill, on the other hand, watched the Food Network when she was unable to eat. Mary confessed to frequent crying episodes in the early days of her disease, but said it made her feel better afterward. Larry described how he used humor to deal with nonempathic peers as part of their coping mechanisms:

“When people are like why, what's wrong with you? I say I have a jacked-up colon.”

Craig and Mary endorsed the use of medical marijuana but expressed reservations in disclosing this to others without knowing their stance on medical marijuana. Mary stated:

“I also smoked marijuana a lot to help with all the pain, it helped me so much. It was actually something I felt sometimes awkward about, because I didn't want to tell my friends as I was just meeting them, because I didn't know their stance on it.”

#### Facing disease challenges

Three students talked about the difficulty of achieving disease remission and multiple medication changes with no assurance about results. They talked about the struggle of meeting their academic goals while dealing with a disease “flare.” Mary, who described herself as a naturally open person, felt embarrassed about the prospect of discussing her disease with others because of its obvious reference to bowels. Amy describes her trepidation in meeting new people.

“Also meeting new people.. [who].. don't know what this disease is and they don't know you yet, so they don't know you without the disease, … the girl who has Crohn's. It kind of defined me at first. So, meeting new people and trying to find my place there was difficult. Just trying to separate myself from the Crohn's was hard.”

Students talked about weight loss due to IBD and the negative criticism from peers and family that followed it. Mary talked more in detail about extreme weight fluctuations, with weight loss brought about by IBD and weight gain with steroids, followed by weight loss again. These changes were hard on her self-esteem and she developed issues related to body consciousness. She also felt guilty about being “skinny” without any effort, compared to her peers' efforts with diet and exercise. Amy shared that:

“Every time I go home, everyone's like, get on the scale, what do you weigh? It's almost become like an eating disorder.”

##### Taking time for family and friends to adjust

Patients' families and friends also mentioned the importance of having time to adjust to the IBD needs and limitations imposed on their son's and daughter's lifestyle choices. Amy mentioned that, at some level, her family was also going through the patient's experience and would likely benefit from support networks. Mary reported her parents' reaction to her IBD.

“I think my parents have a lot of guilt. They keep saying, I wish it was me, I would do anything to have to deal with it instead of you.”

##### Unexpected disease challenges

Students diagnosed close to or after college transition reported having far more unexpected disease challenges because the new diagnosis compounded new academic and personal responsibilities. Amy and Jill said “everything was unexpected.” Most students were taken by surprise by college stress and its effects on their IBD. Amy explained that:

“Between the food and going to the doctor, everything from the moment I got diagnosed to now was unexpected. I never knew what to expect, so it's all kind of strange and different.”

### Findings from reflection papers

The quantitative analysis of the 48 journal entries showed that IBD affected the social life of the students 66% of the time and their ability to engage in coursework 54% of the time.

#### Impact on social life

One participant whose social life was affected 66% of the time described having “to leave a social gathering multiple times …due to the meal that we ate before.” Another participant described an awkward situation in which she was unable to cancel plans with friends in time due to an unexpected flare and “just had to go along although I surely didn't feel like socializing.” One student whose social life was impacted 100% of the time wrote, “I did not do anything social except go to the library and grocery shopping.”

One student described interacting with an unsympathetic waitress at a restaurant dinner while out with family. The need to modify her order prompted significant embarrassment. Asking questions about details of ingredients in the meals were received with impatience. Another student described abdominal discomfort and diarrhea precipitated by frozen yogurt, compelled to stay in close proximity of her dormitory bathroom.

Students wrote about the persistent abdominal distress that impacted their social life. One student wrote that, “IBD has made me self-conscious about my bathroom habits and has prevented me from always feeling comfortable when I'm at social events.”

#### Impact on coursework

Students expressed their personal struggles as they tried to balance disease needs and educational deadlines during periods of high stress such as exams and acknowledged that at times school work could not take priority. “Overall, I learned to listen to my body and not fight what it needs.” Another participant wrote about making difficult choices while missing “a lot of class for doctor appointments.”

Students described in exquisite detail the daily inconveniences caused by their disease such as leaving “the library because I did not want to use their toilet paper for a bowel movement.” A student whose coursework was affected 100% of the time complained that she could not “do as much work” because of persistent pain and prolonged time she had spent in the bathroom. Another student wrote, “Pain and exhaustion affect my concentration so coursework has been a battle.” Another student reported becoming “fatigued and tired [after spending 90 minutes] on the phone with my specialty pharmacy and a related co-pay assistance program trying to solve” new prescription issues.

## Discussion

The main finding of our study is identification of transition to college being a specifically challenging time for students with IBD with interactions with physicians, teachers, and peers playing a critical role in their ability to cope with the disease. IBD impacts student's social life and requires them to develop coping strategies while learning to manage their disease. Students diagnosed nearer to, or after, college transition experience greater difficulty with stress invariably worsening disease symptoms. Overall, students expressed some dissatisfaction over a lack of uniformity in physician guidance regarding diet although descriptions of common beneficial or poorly tolerated diets for IBD patients have been described by others.^13,14^ The rapid weight fluctuations exacerbated by medication side effects resulting in poor body image have been described previously by Knowles et al.^15^ and McDermot et al.^16^ Most students expressed initial frustration with their inability to indulge in conventional college activities and the limitations placed on their social life due to IBD but were appreciative of support from family and friends. Students modified their choices with regards to eating and drinking, but moderating alcohol consumption posed a challenge.

Our study highlights the importance of student support resources on campuses. Disability resource and student health centers at many universities are available to assist students with IBD but often poorly equipped or inexperienced with respect to IBD-related issues to assist student-patients with IBD. Most of our study participants chose to follow a local gastroenterologist in their college town, independently, or in collaboration with their primary hometown gastroenterologist. This highlights the importance of reliable and disease-related health care accessibility for students navigating college with chronic disease. It is important for schools to consider needs of students with IBD, who may not have a visible disability but require support to be successful. Home-town physicians can contribute to the college success of their patients with IBD by assisting with a connection to a local gastroenterologist and directly discussing college transition. Authentic conversations about stress and symptom management, dietary modifications, medication self-management, body image, advice about alcohol and caffeine use, and family education for support can be helpful. Most importantly, there is a need to recognize students' struggles with a disease, which is painful, unpredictable, and poorly understood by their peers and teachers. University support groups can be a medium for acceptance, as well as a valuable forum for encouragement for college students with IBD, who are pursuing higher education in the face of debilitating chronic illness. Stress, negative emotions, and maladaptive thinking have been shown to deleteriously influence gut function through the interaction between the brain's emotional motor system and the autonomic nervous system.^13^ Schwenk et al.^6^ recommended that students with IBD utilize a faculty mentor, a nurse navigator, or health service liaison to provide self-care programs tailored to their preferences and academic demands to promote engagement with university personnel.

Our study has some limitations. Findings from FGs are contextual, temporal, and related only to those individuals that participated. The number of questions that could be asked and the available response time for any participant to answer each question were necessarily limited to hear from everyone. Saturation of data may also have been achieved since our participants were from the same institution and had similar experiences. Students who enrolled in the study were likely to have been more comfortable and forthcoming discussing their struggles with IBD and college transition compared to students with IBD who chose not to participate. The findings are also limited to students' immediate experiences in college rather than their experiences before entry.

## Conclusion

In conclusion, using FGs, we identified factors impacting college-aged IBD patients' college experience, related challenges, and the coping mechanisms they used to manage self-care. Our findings offer guidance for improving students' college success, quality of care, and enhancing physician–patient interactions.

## Abbreviations Used

CD: Crohn's disease
FG: focus group
IBD: inflammatory bowel disease
SD: standard deviation
UC: ulcerative colitis
